# Femoral Component Malrotation Following Total Knee Arthroplasty in the Presence of Hypoplasia: Is the Orthopedic Surgeon Aware?

**DOI:** 10.7759/cureus.75488

**Published:** 2024-12-10

**Authors:** Çağdaş Pamuk, Ülker Moralar, Ümit Gök

**Affiliations:** 1 Orthopaedics and Traumatology, Silivri Anadolu Special Hospital, Istanbul, TUR; 2 Orthopaedics and Traumatology, Kocaeli State Hospital, Kocaeli, TUR

**Keywords:** anterior knee pain syndrome, knee osteoarthritis, knee prosthesis, lower extremity deformities, total knee replacement

## Abstract

Background

This study hypothesizes that patients with femoral condylar hypoplasia who undergo total knee arthroplasty (TKA) may experience femoral component malrotation and that the surgeon performing the operation may not notice it. The aim is to measure the rotational alignment of the femoral components and assess the functional outcomes in these patients.

Materials and methods

Between December 2018 and December 2022, a total of 96 knees from 80 patients were evaluated. The patients were divided into two groups: one with femoral condylar hypoplasia and one without. Rotational changes and functional outcomes were assessed and compared.

Results

The study included 24 patients, 12 in Group 1 and 12 in Group 2. The demographic characteristics of the patients were homogeneous. The duration of knee prostheses ranged from two to four years. Femoral condylar hypoplasia was detected in 15% of all patients. All patients with hypoplasia had femoral components in varying degrees of internal rotation. There was no significant difference in functional scores between the two groups.

Conclusions

The rotation of the femoral component in TKA using the posterior condylar axis is affected by deformities in the condyles. In patients with lateral condylar hypoplasia, using the standard method inevitably results in internal rotation of the femoral component.

## Introduction

Total knee arthroplasty (TKA) is frequently performed as a salvage method in the treatment of osteoarthritis (OA) today [1]. Although TKA may appear to be a bone surgery at first glance, the fundamental principle of the surgical technique lies in perfect soft tissue management. For proper bone cuts, the anatomical, physiological, and biomechanical features of the knee must be thoroughly understood [2]. The rotation and alignment of components directly affect soft tissue balance. Therefore, planning for TKA should involve correcting bone deformities and establishing soft tissue balance simultaneously [3].

If a deformity such as distal femoral hypoplasia is present, using standard TKA reference systems may lead to rotational anomalies, negatively impacting functional outcomes. Successful outcomes depend on accurate bone cuts and proper soft tissue balance [4,5].

Preoperative detection of possible lateral condyle hypoplasia may not always be possible. In such cases, the femoral component may be positioned in internal rotation during TKA. The posterior reference system is commonly used to ensure correct rotational alignment of the femoral component during TKA [6,7].

This study hypothesizes that femoral components may be in internal rotation in patients with femoral condylar hypoplasia who undergo TKA, and the surgeon may be unaware of this malrotation postoperatively.

The aim of this study is to retrospectively identify patients with femoral condylar hypoplasia who underwent TKA, measure femoral component rotations, evaluate the presence of malrotation, and assess their clinical functional outcomes.

## Materials and methods

In this study, 96 knees from 80 patients who underwent TKA by the same surgeon and team at the Orthopedics and Traumatology Clinic of Private Silivri Anadolu Hospital were retrospectively evaluated between December 2018 and December 2022. Ethical approval was obtained from the Medipol University Ethics Committee (No: E-10840098-992.02-2365). Written informed consent was obtained from all patients.

Inclusion and exclusion criteria

The study included patients who underwent TKA for gonarthrosis during the specified period. Patients with the following conditions were excluded from the study: underwent a second surgery on the same knee after TKA, presence of preoperative extension issues (recurvatum, flexion contracture), additional surgical procedures on the tibia or femur during or after component placement, inadequate preoperative lateral radiographs according to the Dejour classification, and did not provide written consent (Table 1).

**Table 1:** Inclusion and exclusion criteria TKA: total knee arthroplasty

Inclusion Criteria	Exclusion Criteria
Patients who underwent TKA for gonarthrosis during the specified period	Patients who underwent a second surgery on the same knee after TKA
Patients who provided written informed consent	Presence of preoperative extension issues (recurvatum, flexion contracture)
Patients with Types C and D dysplasia according to the Dejour classification	Additional surgical procedures on the tibia or femur during or after component placement
	Inadequate preoperative lateral radiographs according to the Dejour classification
	Patient involved in another clinical trial

**Figure 1:**
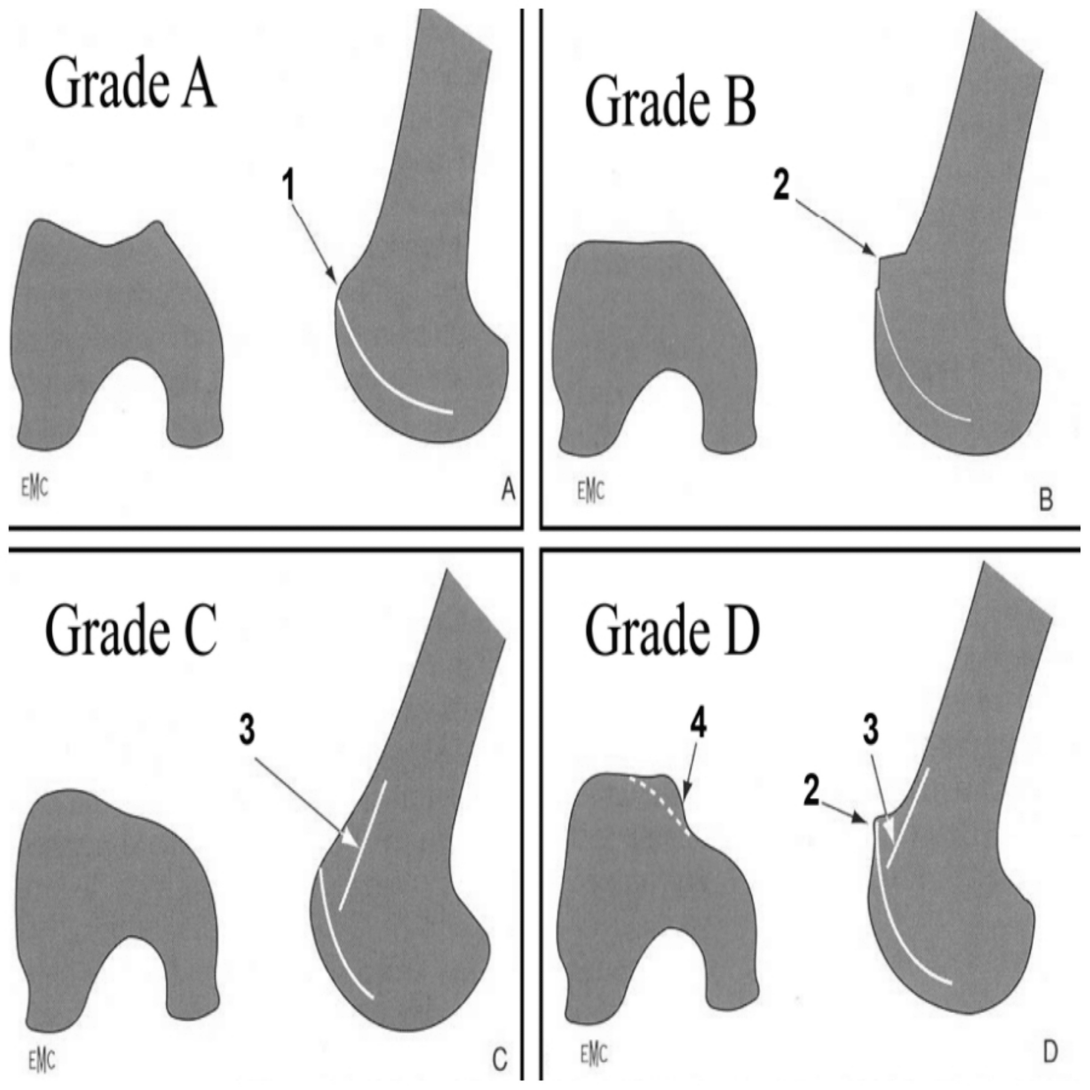
Dejour classification Type A (1) crossing sign, Type B (2) supratrochlear bump or spur, Type C (3) double contour, and Type D (4) vertical join or cliff pattern [8].

According to the Dejour classification, 15 patients with trochlear dysplasia and lateral femoral condylar hypoplasia (Types C and D) (Figure 1) were identified [8]. One patient was excluded due to extra-articular deformity, another due to loss to follow-up, and one declined to provide consent. A total of 24 patients were included, with 12 patients with femoral condylar hypoplasia randomized to Group 1 and 12 patients without deformity to Group 2 (randomized using www.random.com). Preoperative lateral knee radiographs classified 12 knees with Type C or Type D femoral condylar hypoplasia into Group 1. Another 12 knees without hypoplasia were randomized to Group 2. Patients were invited to participate by phone, informed about the study, and provided consent.

**Figure 2:**
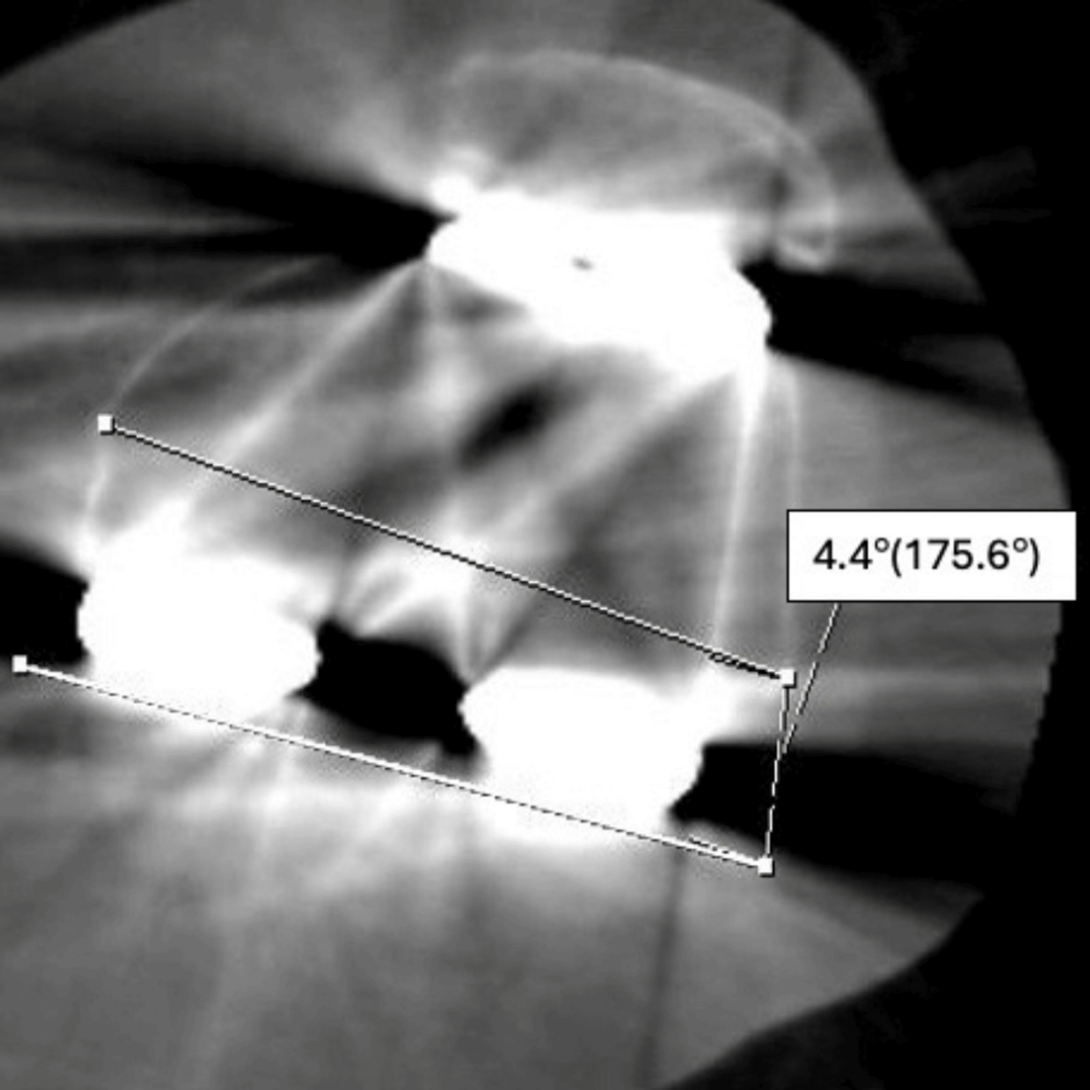
Patient from Group 1 internal rotated femoral component on computed tomography

To measure femoral component rotation, the method described by Berger et al. [10] was used, employing computed tomography (CT) (Siemens Healthcare 2020© Somatom 128 Scanner, PA, USA). The knee was fully extended with a support placed under the heel, and the limb was scanned from the proximal to distal component with 1.5 mm slices. The CT slices were adjusted to pass perpendicularly through the sagittal and coronal planes of the femoral component (Figure 2). The angle between the surgical epicondylar axis and a line passing posteriorly to the component was measured to determine femoral component rotation [9,10].

Patient data, including age, sex, body mass index (BMI), and clinical outcomes, were recorded. Total joint range of motion, femoral component rotation, planned versus actual femoral component rotation, and functional scores using the International Knee Documentation Committee (IKDC) and Western Ontario and McMaster Universities Osteoarthritis Index (WOMAC) were compared [11,12].

Surgical method

Preoperative prophylactic antibiotics (first-generation cephalosporin 1000 mg intravenously, administered one hour before surgery) and deep vein thrombosis (DVT) prophylaxis (low molecular weight heparin: enoxaparin 0.4 ml subcutaneously once daily for one month) were applied to all patients. Surgeries were performed under general or epidural anesthesia. During the procedures, a pneumatic tourniquet (Medione© Trq-2017 Model 128, Turkey) was applied at a pressure of 350 mmHg above the knee level.

All surgeries were conducted by the same surgeon using an anterior longitudinal approach. Cemented, fixed-bearing implants (Smith & Nephew© LEGION Total Knee System, USA) were used in all patients. The posterior cruciate ligament was resected, and the patellar surface was not resurfaced. All patients underwent patellar neurotization. An intraoperative decision was made for lateral release. Posterior condylar referencing was used for femoral cuts, and the guide was set to provide 3° of external rotation. Postoperatively, vacuum drainage and elastic bandages were applied, and patients with no functional or wound-related complications were discharged on the third day.

Statistical analysis

Statistical analysis was performed using IBM SPSS Statistics for Windows, Version 20 (Released 2011; IBM Corp., Armonk, New York). The normality of data distribution was assessed using the Kolmogorov-Smirnov test. Numerical variables were presented as mean ± standard deviation, median (25th-75th percentiles), and frequency (percentages). Differences between groups were analyzed using the Mann-Whitney U test for non-normally distributed numerical variables and Fisher's exact and Yates chi-square tests for categorical variables.

## Results

Between 2018 and 2022, a total of 96 knees from 80 patients undergoing TKA were evaluated according to the Dejour classification. The study included 24 patients: 12 in Group 1 and 12 in Group 2. In Group 1, 66.6% (n=8) of patients were female, and 33.3% (n=4) were male. The operated knee was on the right in 58.3% (n=7) and on the left in 41.6% (n=5) of the cases. The follow-up period ranged from 24 to 56 months (mean = 38 ± 12.4 months). The patients' ages ranged from 52 to 83 years, with a mean age of 69.4 (±16.6). The mean knee flexion was 86.64°. The average IKDC score was 48.8 ± 4.8, and the mean WOMAC score was 38.6 ± 10.6.

In Group 2, 58.3% (n=7) of patients were female, and 41.6% (n=5) were male. The prosthesis was in the right knee in 66.6% (n=8) and the left knee in 33.3% (n=4). The patients' ages ranged from 57 to 69 years, with a mean age of 64.2 (±4.8). The mean knee flexion was 88.6°. The average IKDC score was 54.8 ± 4.7, and the mean WOMAC score was 32.4 ± 6.6.

The incidence of femoral lateral condylar hypoplasia in the study population was 15%. There was no statistically significant difference in knee flexion degrees or femoral rotation between Groups 1 and 2 (p > 0.05).

In Group 1, all femoral components were internally rotated between 1.6 and 4.8° (mean = 2.4 ± 1.6). In Group 2, external rotation ranged from 2.2 to 3.9° (mean = 2.7 ± 1.1). A significant difference in rotation degrees was observed between the two groups (p < 0.001).

No statistically significant difference was found between the IKDC scores and femoral rotation degrees in Groups 1 and 2 (p = 0.568). Similarly, no significant difference was observed between WOMAC scores and femoral rotation degrees (p = 0.195) (Table 2).

**Table 2:** Demographic characteristics of the patients ^a^P-value from Student's t-test ^b^Pearson chi-square test ^c^Mann-Whitney U test f:m, female:male; IKDC, International Knee Documentation Committee; WOMAC, Western Ontario and McMaster Universities Osteoarthritis Index

Demographic Characteristic	Group 1	Group 2	P-value
Age	69.4 ± 16.6	64.2 ± 4.8	.282^a^
Sex (f:m)	8:4	7:5	.604^b^
Body mass index	25.6 ± 2.4	26.1 ± 2.8	.434^a^
Dejour classification			
A	-	8	.762^b^
B	-	4	-
C	3	-	-
D	9	-	-
IKDC score	48.8 ± 4.8	54.8 ± 4.7	.165^a^
WOMAC total score	38.6 ± 10.6	32.4 ± 6.6	.206^c^

## Discussion

TKA is a widely used surgical technique for advanced knee OA, with long-term successful outcomes reported in large series. Achieving proper mechanical axis alignment and stability of the lower extremity is critical for both functional outcomes and prosthesis longevity [1,2,4,5,13]. This study demonstrated that femoral condylar hypoplasia may go unnoticed during standard preoperative planning, even by experienced surgeons. When TKA is performed using standard posterior referencing cutting guides, the femoral component is placed in internal rotation in nearly all cases of hypoplasia [4].

While no significant differences in functional outcomes were observed during follow-up, internal malpositioning of the femoral component is considered an unacceptable surgical error. Functional results after TKA may vary. This is a situation that can be affected by many factors. However, we think that the internal rotation position may have adverse results in the long term. Rand et al. evaluated 11,606 TKA cases in terms of prosthesis survival, reporting survival rates of 91% at 10 years, 84% at 15 years, and 78% at 20 years. They found higher survival rates in patients over 70 years compared to those under 50 [14]. In our series, with a mean follow-up of 38 ± 12.4 months, prosthesis survival was 100%.

The increasing number of TKA procedures has led to a rise in complications and revisions. Approximately 3.3% of all TKA surgeries require revision, with the most common reason being mechanical alignment issues (40%), followed by infection (24%), loosening (9%), instability (9%), and periprosthetic fractures (4%) [2,15,16]. We encountered no revision-related complications in our study. Many authors consider misalignment to be the primary cause of loosening in TKA. Poor femoral rotational alignment can lead to complications such as patellar subluxation, dislocation, and wear, potentially causing anterior knee pain [17].

Common causes of malrotation include surgical technique errors, lack of experience, and inaccuracies in the reference system used during surgery. Femoral condylar hypoplasia, despite meticulous surgical technique, can result in poor outcomes due to rotational alignment challenges. The incidence of lateral femoral condylar hypoplasia is not well documented in the literature. In our study, its incidence was 15%. Conditions associated with femoral condylar hypoplasia include valgus deformity, discoid meniscus, trochlear dysplasia, and knee OA [18-21].

Hypoplastic lateral condyles exhibit increased axial height differences between the lateral and medial condyles, with a narrower posterior condyle in the sagittal plane. Normal knees have an average lateral femoral condyle height of 68 mm (±5.5 mm) and medial height of 60 mm (±5 mm). The posterior offset is approximately 26 mm (±2.2 mm) laterally and 29 mm (±2.8 mm) medially. Hypoplastic condyles present with smaller measurements [8,19,22,23].

During TKA, using posterior condylar referencing systems may result in undesirable internal rotation due to these deformities. The standard 3° external rotation set by cutting blocks may be insufficient or even lead to internal rotation of the femoral component. In our study, all femoral components in Group 1 were internally rotated. We believe these findings support our hypothesis [21,24].

In cases of varus deformity, a deficiency in the posterior medial femoral condyle can cause the surgeon to place the femoral component in excessive external rotation when using the posterior condylar axis as a reference [7]. However, consistent internal rotation in all cases suggests an additional influencing factor, which we believe is the hypoplastic lateral condyle.

Malrotation is often associated with patellofemoral issues. Previous studies have shown that femoral components placed in internal rotation of 4-10° can cause patellofemoral problems, which may be managed by lateral retinacular release. Internal rotation beyond 10° can lead to patellar dislocation and component failure. In our study, no such alignment problems were observed. Femoral components in Group 1 were internally rotated by an average of 2.4 ± 1.6°, while those in Group 2 were externally rotated by an average of 2.7 ± 1.1°. Intraoperatively, patellar and extensor mechanism movements were evaluated, and lateral retinacular release was performed when necessary [3].

Studies have reported that femoral components rotated more than 3° internally or externally negatively affect functional outcomes and reduce knee range of motion [5,10,13]. In our study, there was no significant difference in WOMAC and IKDC scores between Group 1 (internal rotation) and Group 2 (external rotation). We believe the absence of advanced malrotation explains the lack of functional impact, as noted by Berger et al. [10].

Existing evidence suggests that posterior referencing systems can lead to significant errors, causing either internal or external malrotation depending on the deformity's location and shape. Our findings indicate that posterior condylar referencing and standard 3° external rotation cutting blocks may not provide accurate results and are not suitable references. We believe a new reference system and corresponding cutting blocks should be developed [21,24].

The main limitation of our study is its retrospective design. However, as it aims to raise awareness among orthopedic surgeons, a prospective design was not feasible.

## Conclusions

The instruments used during TKA are affected by the patient's existing deformities, potentially leading to unintended outcomes. Instead of applying a standard prosthesis and surgical technique to all cases, using custom cutting blocks tailored to individual cases may yield better results, especially in deformed knees.

The commonly used posterior condylar axis reference system can cause rotational issues in femoral component placement. In patients with lateral condylar hypoplasia, using standard methods inevitably results in internal rotation of the femoral component. There is a need for future randomized controlled studies with larger sample sizes to further explore this issue.
